# Investigating amygdala nuclei volumes in military personnel with post-traumatic stress disorder, major depressive disorder, and adjustment disorder: A retrospective cross-sectional study using clinical routine data

**DOI:** 10.1371/journal.pone.0317573

**Published:** 2025-01-16

**Authors:** Thiemo Knaust, Dagmar Tarnogorski, Matthias B. D. Siebler, Philipp Skiberowski, Christian Moritz, Helge Höllmer, Holger Schulz

**Affiliations:** 1 Center for Mental Health, Bundeswehr Hospital Hamburg, Hamburg, Germany; 2 Department of Radiology, Bundeswehr Hospital Hamburg, Hamburg, Germany; 3 Department of Medical Psychology, University Medical Center Hamburg-Eppendorf, Hamburg, Germany; Idaho State University, UNITED STATES OF AMERICA

## Abstract

**Background:**

Post-traumatic Stress Disorder (PTSD), Major Depressive Disorder (MDD), and Adjustment Disorder (AdjD) are highly prevalent among military personnel, often presenting diagnostic challenges due to overlapping symptoms and reliance on self-reporting. The amygdala, particularly the basolateral complex involved in fear-related memory formation and extinction recall, plays a crucial role in emotional processing. Abnormalities in these amygdala nuclei are implicated in PTSD and may distinguish it from other disorders like MDD and AdjD, where these mechanisms are less central. Investigating structural differences in specific amygdala nuclei could enhance diagnostic precision and inform targeted interventions.

**Goal:**

This study aimed to explore volumetric differences in amygdala nuclei among patients with PTSD, MDD, comorbid PTSD and MDD (PTSD+MDD), and AdjD using routine clinical MRI data. We hypothesized that patients with PTSD would exhibit distinct amygdala nuclei volumes compared to those with MDD or AdjD. Additionally, we examined the influence of symptom duration, prior medication, and psychotherapeutic experience on amygdala volumes.

**Methods:**

We conducted a retrospective cross-sectional study with 185 military personnel (162 men, 23 women) diagnosed with PTSD (n = 50), MDD (n = 70), PTSD+MDD (n = 38), and AdjD (n = 27). High-resolution T1-weighted MRI scans were obtained using a 3T Siemens Skyra scanner. Amygdala subfields were automatically segmented and volumetrized using FreeSurfer software. Analysis of covariance (ANCOVA) models compared amygdala nuclei volumes across diagnostic groups, controlling for estimated total intracranial volume (eTIV), age, and gender. Exploratory analyses included symptom duration, medication use, and prior psychotherapy as additional covariates. Sensitivity analyses further examined the impact of depressive episode type (first vs. recurrent), severity (mild, moderate, severe), and AdjD symptom duration.

**Results:**

The main analyses revealed no significant differences in the volumes of the basolateral and medial amygdala nuclei among the PTSD, MDD, PTSD+MDD, and AdjD groups. Exploratory analyses did not identify significant associations between amygdala volumes and symptom duration, medication use, or prior psychotherapy. Sensitivity analyses also showed no significant volumetric differences related to depressive episode type, severity, or AdjD symptom duration.

**Conclusion:**

Our findings suggest that, within this military population, amygdala nuclei volumes measured using routine clinical MRI data do not significantly differ among patients with PTSD, MDD, PTSD+MDD, and AdjD. This indicates that structural amygdala volumetry alone may not suffice to distinguish between these stress-related disorders in clinical settings. The study highlights the complexity of diagnosing overlapping mental health conditions and underscores the need for comprehensive approaches that integrate neuroimaging with clinical assessments. Future research should include healthy control groups, consider additional brain regions and functional connectivity, and employ longitudinal designs to better understand the temporal dynamics of amygdala changes and their relation to symptomatology.

## 1. Introduction

Mental health disorders such as, Post-traumatic Stress Disorder (PTSD), Major Depressive Disorder (MDD), and Adjustment Disorders (AdjD) are highly prevalent in military populations, posing significant challenges to personal well-being and operational readiness [1]. Traditionally, diagnosing these conditions relies heavily on patients’ introspective abilities and their capacity to verbalize their experiences, which can be challenging due to the nature of these disorders [2]. Such reliance may hinder accurate diagnosis and effective treatment planning. To address this limitation, incorporating additional medical data, such as magnetic resonance rmaging (MRI) scans, holds promise for enhancing diagnostic accuracy. By leveraging routinely collected clinical data, we can improve the differentiation between these disorders, aligning with the principles of precision psychiatry—a field focused on tailoring diagnosis and treatment to individual biological characteristics [3].

While several brain regions are implicated in PTSD and MDD, the amygdala has received specific attention due to its role in emotion regulation, fear conditioning, and memory processing [4,5]. Shalev et al. [6] highlight that abnormalities in the basolateral complex of the amygdala—which is crucial for fear-related memory formation and extinction recall—are particularly associated with PTSD, contributing to persistent fear responses and impaired safety learning [6]. These mechanisms may differentiate PTSD from other disorders like MDD or AdjD, where these processes are less central. Studies comparing amygdala volumes in PTSD [7,8] and MDD [9,10] patients to healthy controls (HCs) have yielded variable results. Some studies suggest a trend towards smaller amygdala volumes, but this is not consistently supported, especially when different comparison groups and methodologies are considered. Specific amygdala nuclei, such as the basolateral nuclei, have been proposed to be associated with distinct symptom clusters in PTSD, but evidence remains mixed.

## 2. Background

### 2.1. PTSD and volumetry of amygdala

PTSD, a severe condition triggered by traumatic events, involves intrusive experiences, hyperarousal, and avoidance behaviors [11]. Research on amygdala volumes in PTSD shows mixed results. For example, O’Doherty et al. [8] reported smaller amygdala volumes in PTSD patients compared to HCs (Hedge’s *g* = -0.50, *p* < .05) but found no significant differences when considering the left (Hedge’s *g* = -0.60, *p* = .101) and right hemispheres separately (Hedge’s *g* = -0.41, *p* = .228), or when comparing PTSD to trauma-exposed healthy controls (TEHC) (Hedge’s *g*_total_ = -0.04, *p* = .673; Hedge’s *g*_left_ = -0.07, *p* = .596; Hedge’s *g*_right_ = -0.05, *p* = .734) [8]. This raises questions about whether these reductions are specific to PTSD or indicative of general stress exposure.

Logue et al. [7] attempted to reduce inconsistencies by using standardized protocols and incorporating unpublished data, but found that the slight reduction in amygdala volume in PTSD patients (effect size *d* = −0.11, *p* = 0.025) compared to a control group consists of HC and TEHC was not significant after correcting for multiple comparisons [7]. This emphasizes the variability of findings based on control group selection [7,8].

#### 2.1.1. Volumetry of the amygdala nuclei in PTSD

The amygdala is composed of nine nuclei, grouped into three primary subregions: basolateral (BLA), centromedial (CMA), and superficial (SFA) [12,13]. The basolateral complex, the largest, includes nuclei crucial for fear-related memory formation, integral to PTSD pathology [6]. The CMA are involved in behavioral responses to emotional stimuli, while the SFA are associated with social and affective processing [12].

More refined insights into PTSD may come from examining individual amygdala nuclei. Morey et al. [14] found that, compared to TEHC, PTSD patients had smaller volumes in the lateral nucleus (Cohen’s *d* = 0.11 to 0.16, *p* = .007 to *p* = .016) and paralaminar nucleus (Cohen’s *d* = 0.25, p = .016 to *p* = .006), and larger volumes in the central (Cohen’s *d* = -0.18 to -0.23, *p* = .001 to *p* = .009), medial (Cohen’s *d* = -0.20 to -0.31, *p* = .001 to *p* = .015), and cortical nuclei (Cohen’s *d* = -0.20 to -0.21, *p* = .001 to *p* = .015) [14]. Similarly, Zhang et al. [15] studied earthquake survivors and found significant volume reductions in both PTSD patients and TEHC compared to HCs, with η^2^ ranging from .007 to .191 and p-values ranging from .049 to < .001. Specifically, they reported significant reductions in the right amygdala (η^2^ = .051, *p* = .006) in both PTSD and TEHC groups compared to HCs. Notably, the left medial nucleus (Me) of the amygdala was significantly larger in PTSD patients than in TEHC (η^2^ = .191, *p* = .027), suggesting that specific nuclei could differentiate PTSD from general stress exposure [15].

These findings suggest that structural differences in specific amygdala nuclei may be associated with PTSD. While Morey et al. [14] observed reductions in the lateral nucleus—which is part of the basolateral complex—in PTSD patients, supporting the model by Shalev et al. [6] that emphasizes the role of the basolateral complex in fear conditioning and extinction processes, the observed enlargements in the medial nucleus reported by both Morey et al. [14] and Zhang et al. [15] may indicate the involvement of additional amygdala regions. This implies that, beyond the basolateral complex, the medial nucleus could also play a significant role in the pathophysiology of PTSD. Based on these findings, although the evidence is still preliminary and more indicative than conclusive, it can be considered that structural differences in amygdala nuclei might also be found in other mental disorders that are less directly involved with fear conditioning and extinction processes [6,14,15].

### 2.2. MDD and volumetry of amygdala

MDD is marked by persistent sadness, hopelessness, and lack of interest, affecting daily functioning and relationships [10,16]. Neuroimaging studies link MDD to differences in brain regions, notably the amygdala [17], but findings are inconsistent. Meta-analyses and reviews suggest a correlation between MDD and reduced amygdala volumes compared to HCs [18], but results vary. Nolan et al. [10] found smaller volumes in some studies (mean Cohen’s *d*: -0.7), while others reported no significant differences [10,19]. Only a few studies examined amygdala nuclei specifically, indicating a need for focused research on potential volumetric differences in MDD.

#### 2.2.1. Volumetry of the amygdala nuclei in MDD

Research on amygdala nuclei volumes in MDD has yielded mixed results. Roddy et al. [20] compared 80 MDD patients to 83 HCs and found that MDD patients had a significantly larger right medial nucleus volume (*p* = 0.002) and increased right-to-left volume ratios in the whole amygdala (*p* = 0.004) and specific nuclei, including the laterobasal composite (*p* = 0.009), central nucleus (*p* = 0.003), and medial nucleus (*p* = 0.014) [20]. Conversely, Kim et al. [21] studied 147 MDD patients and 144 HCs and reported that MDD patients had significantly reduced volumes in the right whole amygdala (*p* < .001), right lateral nucleus (*p* < .001), and right anterior amygdaloid area (*p* < .001) compared to HCs [21]. Other studies, such as those by Brown et al. [16] (n = 24 MDD, n = 20 HCs), Tesen et al. [22] (n = 76 MDD, n = 77 HCs), and Chen et al. [23] (n = 27 MDD, n = 27 HCs), reported no significant differences in amygdala nuclei volumes between MDD patients and HCs [16,22,23]. For instance, Brown et al. [16] found no evidence supporting differences in nuclei volumes after adjusting for age and gender. Tesen et al. [22] observed no volumetric differences but noted inverse associations between depression severity and volumes of the right lateral nucleus and anterior-amygdaloid regions (*p*-values not significant after correction). Similarly, Chen et al. [23] reported no significant volumetric differences. These inconsistencies across studies may be attributed to factors such as sample size, demographic differences, illness duration, first-episode versus recurrent episodes, symptom severity, medication use, and comorbidities.

### 2.3. Volumetry of the amygdala in PTSD with comorbid MDD

MDD frequently occurs alongside PTSD, with a meta-analysis showing that about 52% of individuals with PTSD also have MDD, particularly in military settings [24,25]. Patients with comorbid PTSD and MDD (PTSD+MDD) may show more pronounced volumetric differences in brain structures than those with either condition alone. Yuan et al. [26] found distinct connectivity patterns in PTSD+MDD patients using resting-state functional Magnetic Resonance Imaging (rs-fMRI), indicating that the neural pathophysiology may differ from PTSD or TEHC [26]. While these connectivity patterns provide insight, their relevance to volumetric differences in cardiac magnetic resonance imaging (cMRI) is debated, necessitating caution when translating these findings into structural contexts.

### 2.4. Volumetry of the amygdala in adjustment disorders

AdjD involves an excessive or maladaptive response to stressors, with symptoms typically less severe than those of PTSD or MDD [27,28]. Given its lower severity and shorter stress exposure, AdjD may show fewer alterations in amygdala nuclei. Shalev et al. [6] noted the role of the basolateral complex in fear-related memory formation, suggesting that AdjD may exhibit less impact on these circuits compared to PTSD. However, this remains speculative, as there are currently no studies examining amygdala nuclei in AdjD.

### 2.5. Summary and derivation of the research questions

Mental health disorders such as PTSD, MDD, and AdjD are prevalent in military populations [1], but diagnosis is challenging due to reliance on patients’ self-reporting. Incorporating MRI data may enhance diagnostic accuracy. The amygdala, crucial for emotion regulation and fear processing [6], shows inconsistent volumetric findings in PTSD and MDD compared to HC and TEHC. This study explores volumetric differences in specific amygdala nuclei among PTSD, MDD, PTSD+MDD, and AdjD patients, hypothesizing larger volumes in AdjD due to lower stress severity. Exploratory analyses will examine the influence of factors such as symptom duration, medication, and psychotherapeutic experience on these volumetric differences.

## 3. Materials & methods

The study design received approval from the ethical review board at the Chamber of Physicians in Hamburg, Germany (Reference No.: PV7098), the Administrative Data Protection Officer of the Bundeswehr Hospital Hamburg, and the research conference of the Bundeswehr Medical Academy Munich, Germany (44 K2-S-322224).

### 3.1. Sample and clinical parameters

The sample comprised N = 185 patients (162 men, 23 women) who underwent MRI examinations as part of inpatient psychiatric treatment at the Bundeswehr Hospital Hamburg from January 2014 to March 2019. The secondary processing of the cMRI scans, along with access to the corresponding medical reports, extended until December 1, 2019. During this period, the data were pseudonymized using patient case numbers. Following the data collection phase, all data were anonymized. Statistical analysis was then performed using the anonymized dataset, making individual participant identification unfeasible.

Inclusion criteria for this retrospective cohort study categorized patients into four groups to test hypotheses: (i) MDD patients (individuals with a single or recurrent major depressive episode in mild, moderate, and severe severity according to ICD-10 criteria, n = 70, 38%), (ii) PTSD-D patients (individuals diagnosed with PTSD without a secondary diagnosis of MDD according to ICD-10, n = 50, 27%), (iii) PTSD+D (individuals diagnosed with PTSD and with a single or recurrent major depressive episode in mild, moderate, and severe severity according to ICD-10, n = 38, 21%), and (iv) AdjD (individuals diagnosed with adjustment disorders according to ICD-10, n = 27, 15%). Additionally, an MRI scan had to be ordered during the inpatient stay. Exclusion criteria included admission for previous intracranial injury; central nervous system disorders such as epilepsy, multiple sclerosis, or intracranial tumors; psychosis; alcohol or drug dependence; and contraindication to MRI. No other inclusion or exclusion criteria were applied.

Patient ages ranged from 18 to 61 years (*M* = 31.96, *SD* = 8.96). Among the participants, 85 (45.9%) had psychopharmacological pretreatment, and 85 (45.9%) had psychotherapeutic pretreatment. A detailed list of the psychopharmaceuticals is provided in Table 1; however, due to the small sample sizes in each medication category, medications were considered dichotomously in the analyses. The reported symptom duration was recorded (*M* = 42.68 months, *SD* = 48.1, range: 1–269 months). The extended durations may be due to patients noticing ’bridging symptoms,’ such as nightmares, early on, even before a full diagnosis of PTSD is established, which should be considered when interpreting the results.

**Table 1:** Soziodemographics.

		MDD		PTSD-MDD		PTSD+MDD		AdjD		Test statistics			
Variables		(n = 70)		(n = 50)		(n = 38)		(n = 27)		Chi^2^ / F	(df)	*p*	*ω* / η_p_^2^
Age										7.0	(3, 181)	< .001	.104
	*M* (*SD*)	29.2	(8.9)	34.3	(8.2)	35.7	(8.1)	29.3	(8.7)				
Gender (male)										1.6	(3)	.640	.095
	*n* (%)	63	(90%)	44	(88%)	31	(81%)	24	(88%)				
Education										6.3	(6)	.390	.132
University	*n* (%)	12	(17%)	9	(18%)	2	(5%)	5	(18%)				
Highschool	*n* (%)	15	(21%)	6	(12%)	8	(21%)	7	(25%)				
Middleschool	*n* (%)	42	(60%)	33	(68%)	28	(73%)	15	(55%)				
Military rank										7.6	(6)	.261	.144
OF	*n* (%)	15	(21%)	10	(20%)	4	(10%)	7	(25%)				
OR 5–9	*n* (%)	32	(45%)	22	(44%)	26	(68%)	11	(40%)				
OR 1–4	*n* (%)	23	(32%)	18	(36%)	8	(21%)	9	(33%)				
Medication										26.0	(3)	< .001	.324
Yes	*n* (%)	37	(52%)	21	(42%)	24	(63%)	3	(11%)				
SSRIs	*n*	14	(52%)	6	(28%)	11	(45%)	1	(33%)				
TZA	*n*	1	(1%)	3	(14%)	1	(4%)	0	(0%)				
Atypical antipsychotic SSNRI	*n* *n*	2 4	(3%) (6%)	0 2	(0%) (9%)	2 2	(8%) (8%)	0 0	(0%) (0%)				
NaSSA	*n*	6	(9%)	2	(9%)	4	(16%)	1	(33%)				
Other psycho.		10	(14%)	8	(38%)	4	(16%)	1	(33%)				
pre psychotherapy										22.8	(3)	< .001	.351
Yes	*n* (%)	25	(35%)	28	(56%)	27	(71%)	5	(5%)				
Symptom duration										12.2	(3, 160)	< .001	.191
	*M* (*SD*)	23.6	(20.3)	61.5	(58.9)	70.4	(59.4)	23.8	(31.7)				

*Note*. AdjD = Patients with adjustment disorders, MDD = Patients with major depressive disorder, M = Mean, NaSSA = Noradrenergic and Specific Serotonergic Antidepressant, OF = Officers, OR 1–4 = Enlisted ranks, OR 5–9 = Noncommissioned officers, PsyPharm pre-treat = Psychopharmacological pre-treatment, PsyT pre-treat = Psychotherapeutic pre-treatment, PTSD-MDD = Patients with posttraumatic stress disorder without comorbid MDD, PTSD+MDD = Patients with posttraumatic stress disorder with comorbid MDD, SD = Standard deviation, SSRI = Selective serotonin reuptake inhibitors, SSNRI = Selective Serotonin Noradrenalin Reuptake Inhibitors, TZA = tricyclic antidepressant.

All information on the patients’ medical history, MRI data, and findings were obtained from the Center of Mental Health and the Department of Radiology of the Bundeswehr Hospital Hamburg. Psychiatric findings were assigned by a specialist in psychiatry following ICD-10 criteria, and a radiology specialist assessed the MRI data. Psychiatric diagnoses were determined through anamnesis and reevaluated during the inpatient stay. Although the patients generally underwent psychometric testing, it was not necessary for diagnosis assignment and was not accessible to us for data protection reasons.

### 3.2. Analysis and MRI data processing

MRI scans were conducted utilizing a 3T Siemens Skyra MRI scanner (Siemens AG Medical Solutions, Erlangen, Germany) at the Department of Radiology of the Bundeswehr Hospital Hamburg. A 20-channel head coil was employed for the imaging process. Structural images of the amygdala were captured through a sagittal three-dimensional T1-weighted gradient echo sequence known as MPRAGE (Magnetization Prepared Rapid Gradient Echo), with the following parameters: TR (Repetition Time) of 2300 ms, TE (Echo Time) of 2.3 ms, matrix size of 256 x 256, voxel size of 0.9 mm^3^, and 192 slices.

For image reconstruction and automated delineation of amygdala subfields, FreeSurfer version 6.0.41 was employed. Grounded in a Bayesian model, FreeSurfer predicted the locations of neuroanatomical structures based on probabilistic atlases and localized manual amygdala segmentation from test participants [13]. The software computed nine amygdala nuclei per hemisphere, and these automated segmentations underwent validation against manual morphometric measurements of ultra-high-resolution scans, showcasing improved comparability across different studies [13].

The FreeSurfer software was utilized to calculate the estimated total intracranial volume (eTIV), providing a measure comparable to manually determined ICV [29]. The inclusion of eTIV as a covariate [30] in our calculations was crucial, considering that certain structures, such as the amygdala, exhibit correlations with the overall head size [31].

### 3.3. Statistical analyses

Statistical analyses were executed using SPSS version 28 software, focusing on four amygdala subfields: Ba, La, AB, and Me, informed by prior research (PTSD: [14,15]; MDD: [20,21]). Due to the absence of hemisphere specificity for the basolateral and medial nucleus complex, left and right hemispheres were amalgamated to compute the total volume. Primary outcome variables comprised the total volume of Ba, La, AB, and Me subfields, with inferential statistics deployed to minimize false positives.

A one-way analysis of covariance (ANCOVA) was conducted for each outcome variable, with the patient group as the independent variable and the target variables (Ba, La, AB, Me) as the dependent variables. eTIV was included as a covariate to account for individual differences in total intracranial volume. Additionally, sociodemographic factors such as age and gender were included as covariates to control for their potential influence on amygdala volumes. When a significant main effect of the patient group was found, a priori planned contrasts using independent sample t-tests were performed to explore volume differences among the PTSD-D, PTSD+D, and MDD groups relative to AdjD. Exploratory, undirected *t*-tests for independent samples were conducted to investigate potential differences between the PTSD-D, PTSD+D, and MDD groups. To address exploratory questions, we extended the one-way ANCOVA by including additional covariates: (i) symptom duration, (ii) medication, and (iii) prior psychotherapeutic experience, along with their interaction terms with the patient group factor.

Inferential analyses focused on the Ba, La, AB, and Me nuclei, while descriptive statistics were computed for all amygdala nuclei to provide a comprehensive overview. Table 2 summarizes the eTIV-corrected means and 95% confidence intervals (CI) of the means. Additionally, eTIV-corrected effect sizes (Cohen’s *d* for unequal-sized samples; [32]) between stress-related mental disorders for each amygdala nucleus, categorized by hemisphere, are presented in Table 3.

**Table 2:** Descriptive statistics of the amygdala nuclei corrected according to eTIV (voxel size: 0.9 mm^3^).

	MDD without PTSD (*n* = 70)			PTSD without MDD (*n* = 50)			PTSD with MDD (*n* = 38)			AdjD (*n* = 27)		
variable	*M*	95% CI		*M*	95% CI		*M*	95% CI		*M*	95% CI	
Left												
AAA	62.5	[60.9,	64.2]	62.3	[60.9,	64.7]	60.6	[58.4,	62.8]	62.7	[60.2,	65.3]
CAT	204.4	[200.1,	208.7]	201.6	[196.6,	206.7]	196.8	[190.9,	202.6]	202.5	[195.7,	209.4]
La	731.4	[716.9,	745.8]	730.3	[713.4,	747.2]	723.3	[703.7,	742.8]	723.0	[700.1,	746.0]
Ba	501.2	[491.0,	511.5]	497.2	[485.3,	509.2]	490.2	[476.3,	504.1]	500.2	[483.9,	516.5]
PL	56.3	[55.2,	57.5]	55.8	[54.4,	57.2]	55.3	[53.7,	56.9]	55.4	[53.5,	57.3]
AB	301.5	[295.2,	307.8]	296.1	[288.8,	303.5]	290.2	[281.7,	298.7]	305.9	[295.9,	315.9]
Me	24.4	[23.2,	25.6]	23.7	[22.2,	25.1]	23.5	[21.9,	25.2]	25.5	[23.6,	27.5]
Ce	50.6	[49.4,	52.3]	48.4	[46.4,	50.3]	49.4	[47.2,	51.6]	53.9	[51.3,	56.5]
Co	29.4	[28.5,	30.2]	28.3	[27.2,	29.3]	28.1	[26.9,	29.3]	29.7	[28.3,	31.1]
Right												
AAA	66.7	[64.9,	68.5]	67.2	[65.1,	69.3]	66.1	[63.6,	68.5]	66.6	[63.8,	69.5]
CAT	209.1	[204.4,	213.8]	206.2	[200.7,	211.7]	202.6	[196.3,	209.0]	209.4	[202.0,	216.9]
La	738.5	[725.0,	752.1]	744.1	[728.2,	759.9]	737.2	[718.8,	755.6]	732.6	[711.0,	754.2]
Ba	510.8	[500.8,	520.8]	508.6	[496.9,	520.2]	500.7	[487.2,	514.3]	509.0	[493.2,	524.9]
PL	55.9	[54.8,	57.0]	55.1	[53.8,	56.3]	54.5	[53.1,	56.0]	55.2	[53.4,	56.9]
AB	312.1	[305.5,	318.7]	312.1	[304.4,	319.8]	305.9	[297.0,	314.9]	315.6	[305.1,	326.1]
Me	27.0	[25.7,	28.3]	26.1	[24.6,	27.6]	26.4	[24.7,	28.2]	28.0	[25.9,	30.0]
Ce	54.7	[53.2,	56.3]	54.0	[52.1,	55.8]	54.6	[52.5,	56.7]	55.6	[53.1,	58.1]
Co	31.2	[30.3,	32.1]	31.2	[30.2,	32.3]	30.7	[29.4,	31.9]	32.5	[31.1,	34.0]
Left + Right												
AAA	129.3	[126.2,	132.4]	130.0	[126.4,	133.7]	130.0	[126.4,	133.7]	129.4	[124.5,	134.3]
CAT	413.5	[405.4,	421.7]	407.8	[398.3,	417.3]	407.8	[398.3,	417.3]	412.0	[399.1,	424.9]
La	1470.0	[1443.4,	1496.5]	1474.4	[1443.4,	1505.5]	1474.4	[1443.4,	1505.5]	1455.6	[1413.4,	1497.9]
Ba	1012.1	[993.4,	1030.8]	1005.8	[984.0,	1027.7]	1005.8	[984.0,	1027.7]	1009.3	[979.6,	1039.0]
PL	112.3	[110.2,	114.4]	110.9	[108.5,	113.4]	110.9	[108.5,	113.4]	110.6	[107.2,	113.9]
AB	613.7	[602.0,	625.4]	608.3	[594.6,	621.9]	608.3	[594.6,	621.9]	621.6	[603.0,	640.1]
Me	51.4	[49.2,	53.6]	49.8	[47.3,	52.3]	49.8	[47.3,	52.3]	53.6	[50.1,	57.0]
Ce	105.4	[102.6,	108.2]	102.4	[99.1,	105.6]	102.4	[99.1,	105.6]	109.5	[105.1,	114.0]
Co	60.6	[59.0,	62.1]	59.5	[57.7,	61.4]	59.5	[57.7,	61.4]	129.4	[124.5,	134.3]

*Note*. AAA = Anterior Amygdala Area, AB = Accessory Basal Nucleus, AdjD = Patients with adjustment disorders, Ba = Basal Nucleus, CAT = Cortico-Amygdaloid Transition Area, Ce = Central Nucleus, Co = Cortical Nucleus, Depr = Patients with depression, eTIV = estimated Total Intracranial Volume, La = Lateral Nucleus, M = Mean value, Me = Medial Nucleus, PL = Paralaminar Nucleus, SD = Standard deviation.

**Table 3:** eTIV corrected between-subjects effect sizes.

	MDD vs. PTSD-MDD		MDD vs. PTSD+MDD		MDD vs. AdjD		PTSD-MDD vs. PTSD+MDD		PTSD-MDD vs. AdjD		PTSD+MDD vs. AdjD	
Variables	Mdiff	Cohen’s *d*	Mdiff	Cohen’s *d*	Mdiff	Cohen’s *d*	Mdiff	Cohen’s *d*	Mdiff	Cohen’s *d*	Mdiff	Cohen’s *d*
Left												
AAA	-0.25	0.03	1.89	-0.27	-0.19	0.02	2.15	-0.31	0.05	-0.00	-2.09	0.30
CAT	2.78	-0.15	7.65	-0.42	1.88	-0.10	4.86	-0.26	-0.89	0.05	-5.76	0.31
La	1.08	-0.01	8.09	-0.13	8.36	-0.13	7.01	-0.11	7.27	-0.12	0.26	-0.00
Ba	3.99	-0.09	11.0	-0.25	1.00	-0.02	7.04	-0.16	-2.98	0.07	-10.01	0.23
PL	0.52	-0.10	1.00	-0.20	0.94	-0.19	0.47	-0.09	0.42	-0.08	-0.05	0.01
AB	5.36	-0.20	11.3	-0.42	-4.37	0.16	5.94	-0.22	-9.74	0.37	-15.69	0.59
Me	0.71	-0.13	0.84	-0.16	-1.15	0.22	0.13	-0.02	-1.87	0.36	-2.00	0.38
Ce	2.28	-0.32	1.25	-0.18	-3.23	0.46	-1.02	0.14	-5.51	0.71	-4.49	0.64
Co	1.08	-0.29	1.25	-0.33	-0.32	0.08	0.16	-0.04	-1.41	0.37	-1.57	0.42
Right												
AAA	-0.48	0.06	0.66	-0.08	0.11	-0.01	1.14	-0.15	0.59	-0.07	-0.55	0.07
CAT	2.92	-0.14	6.43	-0.32	-0.36	0.01	3.51	-0.17	-3.28	0.16	-6.79	0.34
La	-5.52	0.09	1.33	-0.02	5.95	-0.10	6.85	-0.12	11.47	-0.20	4.62	-0.08
Ba	2.21	-0.05	10.04	-0.23	1.76	-0.04	7.83	-0.18	-0.45	0.01	-8.28	0.19
PL	0.83	-0.18	1.40	-0.30	0.77	-0.16	0.56	-0.12	-0.06	0.01	-0.62	0.13
AB	0.02	-0.00	6.16	-0.22	-3.49	0.12	6.13	-0.22	-3.52	0.12	-9.66	0.34
Me	0.89	-0.16	0.55	-0.10	-1.02	0.18	-0.34	0.06	-1.91	0.35	-1.57	0.28
Ce	0.76	-0.11	0.14	-0.02	-0.88	0.13	-0.62	0.09	-1.65	0.25	-1.03	0.15
Co	-0.06	0.01	0.49	-0.13	-1.35	0.35	0.56	-0.14	-1.29	0.33	-1.85	0.47
Total												
AAA	-0.73	0.05	2.56	-0.19	-0.08	0.00	3.29	-0.25	0.65	-0.05	-2.64	0.20
CAT	5.70	-0.16	14.09	-0.40	1.52	-0.04	8.38	-0.24	-4.18	0.12	-12.56	0.36
La	-4.43	0.04	9.42	-0.08	14.31	-0.12	13.86	-0.12	18.75	-0.16	4.89	-0.04
Ba	6.21	-0.07	21.08	-0.26	2.77	-0.03	14.87	-0.18	-3.43	0.04	-18.31	0.23
PL	1.36	-0.15	2.40	-0.27	1.72	-0.19	1.04	-0.11	0.35	-0.04	-0.68	0.07
AB	5.39	-0.11	17.47	-0.35	-7.87	0.15	12.08	-0.24	-13.26	0.27	-25.35	0.51
Me	1.60	-0.17	1.39	-0.15	-2.17	0.23	-0.20	0.02	-3.78	0.41	-3.57	0.39
Ce	3.04	-0.26	1.40	-0.11	-4.12	0.35	-1.64	0.14	-7.17	0.61	-5.55	0.47
Co	1.02	-0.18	1.75	-0.29	-1.67	0.26	0.72	-0.11	-2.70	0.39	-3.42	0.44

*Note*. AAA = Anterior Amygdala Area, AB = Accessory Basal Nucleus, AdjD = Patients with adjustment disorders, Ba = Basal Nucleus, CAT = Cortico-Amygdaloid Transition Area, Ce = Central Nucleus, Co = Cortical Nucleus, Depr = Patients with depression, eTIV = estimated Total Intracranial Volume, La = Lateral Nucleus, M = Mean value, Me = Medial Nucleus, PL = Paralaminar Nucleus, SD = Standard deviation.

Assumptions of normal distribution were assessed using histograms for each patient group. Visual inspection indicated an unclear normal distribution in all cases. Consequently, we repeated the analysis using the bootstrapping procedure (bootstrap samples: *k* = 1,000 with bias-corrected confidence intervals; [33], reporting bootstrapped parameter estimates in S1 Table. No substantial differences were noted. Boxplots facilitated outlier analysis for each target variable and patient group. Analyses of covariance were conducted with and without identified outliers [34]. Importantly, results with and without outliers exhibited no significant differences. S2 Table presents results with and without outliers, while the manuscript conveys findings with outliers.

In addition to the primary analyses, we conducted sensitivity analyses to examine whether the type of depressive episode (first episode vs. recurrent episodes), the severity of depressive episodes (mild, moderate, severe), and the duration of symptoms in AdjD (<6 months vs. >6 months) explained additional variance in amygdala nuclei volumes. To achieve this, we expanded the original ANCOVA models by subdividing the patient groups, transforming the initial four-level factor into a six-level factor for each analysis. The first expanded analysis provided a more nuanced understanding of how episode type in depression and symptom duration in AdjD might affect amygdala nuclei volumes, acknowledging the variability that exists even within single disorder categories. The second sensitivity analysis focused on the severity levels of depressive episodes, categorizing patients according to ICD-10 criteria into mild, moderate, and severe depressive episodes. This allowed us to assess whether the severity of depression had an impact on amygdala nuclei volumes.

#### 3.3.1. Power analysis

A post-hoc power analysis was executed with an adjusted alpha level of 0.0125 and 80% power, involving N = 185 participants, three covariates (eTIV), and a single independent variable (patient groups: MDD, PTSD, PTSD + MDD, and AdjD) for each ANCOVA. The results suggested the ability to detect differences at an effect size of *f*  ≥ 0.28 (with effect sizes characterized as 0.10  =  small, 0.25  =  medium, and 0.40  =  large; [35]). The power analysis utilized G*Power software [36]. Given the four ANCOVAs conducted, we applied the Bonferroni correction, adjusting the alpha level to α  =  0.0125 [33].

## 4. Results

**Fig 1:**
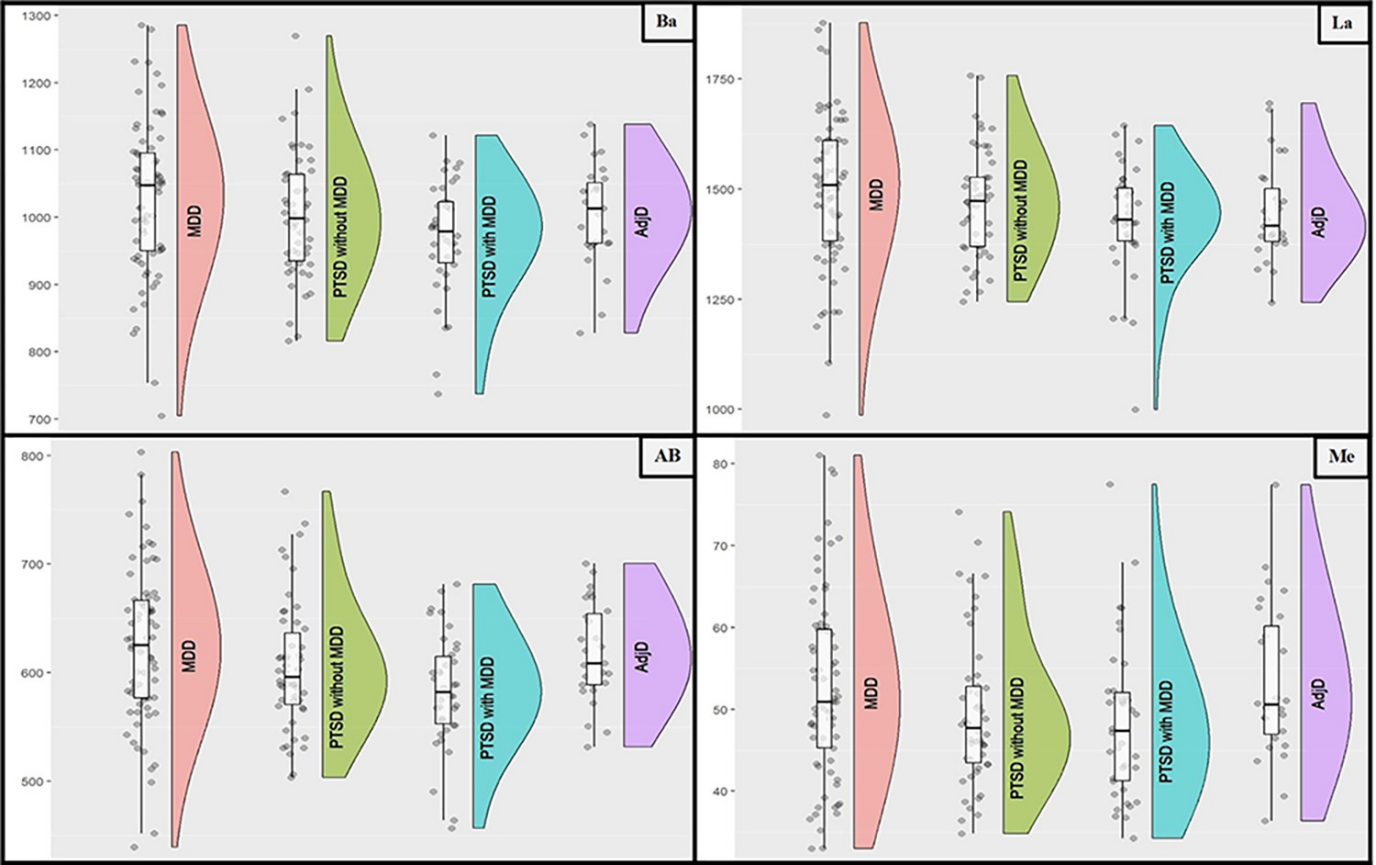
Raincloudplots with boxplots for each independent variable. La = Lateral, Ba = Basal, AB = accessory basal, Me = Medial (voxel size: 0.9 mm^3^).

The outcomes of the ANCOVAs, encompassing both primary and exploratory analyses, are synthesized in Table 4. Additionally, Fig 1 illustrates a raincloud plot supplemented with boxplots, offering a succinct visual representation of the principal findings. The results of these sensitivity analyses are presented in S3–S5 Tables.

**Table 4:** Overview of the ANCOVA results.

	Basal nucleus				Lateral nucleus				Accessory basal nucleus				Medial nucleus			
variables	F	(df)	*p*	η_p_^2^	F	(df)	*p*	η_p_^2^	F	(df)	*p*	η_p_^2^	F	(df)	*p*	η_p_^2^
*Main analyses*																
patient group	0.2	(3, 178)	.872	.004	0.2	(2, 178)	.837	.005	0.7	(3, 178)	.544	0.12	0.7	(3, 178)	.518	.013
*Explorative analyses*																
symptom duration	1.0	(1, 141)	.310	.007	0.6	(1, 141)	.414	.005	1.7	(1, 141)	.183	.013	0.3	(1, 141)	.556	.002
symptom duration^*^patient group	0.9	(3, 141)	.403	.020	1.9	(3, 141)	.132	.039	0.6	(3, 141)	.607	.013	0.4	(3, 141)	.706	.010
medication	0.3	(1, 141)	.543	.003	0.1	(1, 141)	.788	.001	0.2	(1, 141)	.596	.002	0.1	(1, 141)	.772	.001
medication^*^patient group	0.9	(3, 141)	.426	.020	0.1	(3, 141)	.930	.003	1.0	(3, 141)	.352	.023	0.9	(3, 141)	.416	.020
pre psychotherapy	0.6	(1, 141)	.420	.005	0.1	(1, 141)	.671	.001	1.2	(1, 141)	.257	.009	3.7	(1, 141)	.055	.026
pre psychotherapy^*^patient group	0.1	(3, 141)	.924	.003	0.4	(3, 141)	.726	.009	0.6	(3, 141)	.615	.013	0.5	(3, 141)	.665	.011
*Controlling for*																
eTIV	69.0	(1, 178)	< .001	.280	75.0	(1, 178)	< .001	.297	76.0	(1, 178)	< .001	.299	41.4	(1, 178)	< .001	.189
Age	2.9	(1, 178)	.090	.016	0.1	(1, 178)	.745	.001	4.9	(1, 178)	.027	.027	1.7	(1, 178)	.185	.010
Gender	13.6	(1, 178)	< .001	.071	19.4	(1, 178)	< .001	.099	8.4	(1, 178)	.004	.045	0.1	(1, 178)	.795	.001

*Note*. eTIV = estimated intracranial volume, pre psychotherapy = pretreatment psychotherapeutic.

In summary, no significant differences were detected among patient groups for the basal, lateral, accessory basal, or medial nuclei. Furthermore, no significant correlations were observed regarding symptom duration, medication usage, or psychotherapeutic interventions, along with their corresponding interaction effects. Additionally, sensitivity analyses using the six-level ANCOVA, as well as the inclusion of age and gender as covariates, did not significantly alter the results.

## 5. Discussion

We observed no significant differences in the volumes of the basolateral and medial nuclei complexes among patients with MDD, PTSD-MDD, PTSD+MDD, and AdjD. One plausible interpretation of these findings is that the use of routine clinical data for examining amygdala nuclei may not be sufficiently sensitive to differentiate between those disorders. This limitation may stem from challenges in translating research findings into clinical applications, the robustness of previous findings, or constraints inherent in our study methodology.

Some studies have found evidence of smaller amygdala volumes in patients with PTSD and MDD compared to HCs. However, these results remain inconclusive due to considerable heterogeneity and inconsistency in the literature [7,8,14,15,20,21]. Despite these inconclusive findings, various molecular mechanisms have been proposed to explain potential differences, including neuroendocrine, glutamatergic, and inflammatory models [4]. These explanatory frameworks align with findings from rodent studies, which suggest that stress-induced neurotoxicity can result in amygdala atrophy in rats [37,38].

However, it remains uncertain whether this phenomenon is stress-related or disorder-related [4]. The latter would be particularly pertinent in clinical contexts where access to HCs is typically limited. A morphological disease-specific differentiation between PTSD, MDD and AdjD would be especially beneficial within military settings, where those disorders are the most prevalent mental health disorders. In this context, it is valuable to revisit the findings of two previous meta-analyses on PTSD. O’Doherty et al. [8] found a significantly lower amygdala volume only when comparing PTSD patients to HCs, but not when comparing to TEHCs, while Logue et al. [7] reported no significant volume differences at all. These mixed findings underscore the inconclusiveness of the current meta-analytic evidence, particularly given the varying comparison groups used in studies—some involving trauma-exposed and others trauma-non-exposed controls. This variability makes it unclear whether smaller amygdala volumes observed in PTSD and MDD are truly present compared to HCs and, if so, whether they are associated with general stress-related factors or specific to particular disorders.

Some researchers suggest that focusing on the amygdala nuclei could offer a more detailed understanding of its functionality, potentially revealing distinct effects across different mental disorders [4]. Shalev et al. [6] propose a model that highlights the basolateral complex of the amygdala as central to fear learning and extinction recall—key processes involved in PTSD. According to this model, abnormalities in these nuclei could underlie the persistent fear responses and impaired safety learning typical of PTSD. However, the reliance on HCs and cross-sectional study designs in most research limits the interpretation of these findings. It remains uncertain whether volume differences in the basolateral nuclei are specific to PTSD or reflect a more generalized response to stress. Early findings on specific nuclei, such as the basolateral and medial nuclei (PTSD: [14,15]; MDD: [20,21], indicate that their involvement might be related to general stress exposure rather than a disorder-specific mechanism. In our analysis, we found no significant volume differences in the basolateral nuclei among PTSD, MDD, and AdjD groups, further questioning whether the basolateral nuclei can be used as a specific distinguishing feature between these disorders. This underscores the need for more careful selection of comparison groups to better determine whether examining amygdala nuclei can effectively distinguish between different stress-related mental disorders.

Our non-significant findings suggest that reduced amygdala volumes, whether at the total volume or nuclei level, may reflect a generalized response to stress. Under this premise, any distinctions among stress-related mental disorders are anticipated to yield relatively small effect sizes. Thus, it is conceivable that our study may have been underpowered, although our sample size is comparable to previous studies [14,15,20,21].

Despite this, we deliberately incorporated AdjD into our study design, hypothesizing that its typically shorter duration and lower stress exposure severity [27,28], and less pronounced involvement of fear learning and extinction recall processes—mechanisms particularly associated with the basolateral nucleus—compared to PTSD and MDD might result in less pronounced alterations in the basolateral and medial nuclei. Contrary to our expectations, however, we did not observe any significant differences. This lack of distinction could be interpreted as further evidence of an association between reduced amygdala nuclei volumes and a generalized stress response. Nonetheless, it is worth noting that this interpretation remains speculative given the absence of HCs in our study.

In our sensitivity analyses, we explored whether distinctions within diagnostic categories—specifically, between first-episode and recurrent MDD (S3 Table), between mild, moderate, and severe depressive episodes (S4 Table), and between AdjD patients with symptoms lasting less than six months versus more than six months (S3 Table)—would reveal differences in amygdala nuclei volumes. Consistent with our primary findings, these analyses did not demonstrate significant volumetric differences among these subgroups. This lack of differentiation aligns with the inconsistencies reported in the existing literature regarding amygdala volumes in stress-related mental disorders [14,15,20,21].

Our findings also underscore the complexities involved in translating neuroimaging research into practical diagnostic applications. In real-world clinical settings, patients present with a wide array of symptoms, varying durations of illness, and diverse treatment histories. Such heterogeneity makes it challenging to identify consistent neurobiological markers that are robust enough to differentiate between disorders like PTSD, MDD, and AdjD. Even if subtle volumetric differences in the basolateral amygdala exist, they may not be detectable across heterogeneous samples or may not be sufficiently specific to guide diagnostic decisions.

Furthermore, it remains unclear when potential volumetric differences in the amygdala emerge, how they evolve over the course of the illness, or how they respond to treatment [5,17]. The dynamic nature of neurobiological changes necessitates longitudinal studies to elucidate these patterns. In the absence of such data, cross-sectional studies like ours can only provide limited insights.

Given these considerations, our results suggest caution in relying on routine clinical MRI data for the volumetric assessment of the amygdala as a means to differentiate between stress-related mental disorders. The quest for reliable biomarkers must account for the multifaceted and overlapping features of these conditions, as well as the practical realities of clinical populations. Future research should aim for more stringent study designs, including longitudinal assessments, incorporation of multiple control groups (e.g., trauma-exposed and trauma-unexposed healthy controls), and comprehensive evaluations of symptom severity, trauma history, and treatment variables.

Various factors may have influenced morphological differences, including symptom duration [16,21], medication treatment [18], and psychotherapeutic experiences [39]. Consequently, we perform exploratory analyses to examine the relationship between these variables and the volumes of the Ba, La, AB, and Me nuclei, as well as to explore their potential impact on differences observed between stress-related mental disorders.

Prolonged symptom duration may lead to a chronic stress response, potentially reducing amygdala volume [4]. However, findings remain inconclusive, with many studies showing no significant impact of symptom duration. For instance, Ben-Zion et al. [40] conducted a longitudinal study with 100 trauma survivors, where only 29 met PTSD criteria 14 months post-trauma. Their Bayesian analysis found no evidence of time-dependent changes in amygdala nuclei, supporting the vulnerability trait hypothesis and suggesting that significant volume reductions may require longer periods. While some earlier longitudinal studies in MDD observed greater decline in amygdala volume over time compared to controls–specifically in the left amygdala [41]–other studies did not find significant correlations between amygdala volumes and illness duration [42]. In Frodl et al. [41], patients with MDD showed significantly greater gray matter density decline in the left amygdala over a three-year follow-up period compared to healthy controls, whereas the right amygdala did not show such differences. Conversely, in Lange et al. [42] reported no significant correlations between amygdala volumes (left or right) and clinical variables such as illness duration in depressive subjects. Similarly, more recent research at the nuclei level, including our exploratory analysis, found no significant associations between illness duration and amygdala nuclei volumes [16,21]. While earlier studies in MDD indicated correlations between illness duration and amygdala atrophy [41,42], more recent research at the nuclei level, including our exploratory analysis, found no such associations. Future longitudinal studies should explore whether symptom duration correlates with changes in amygdala nuclei and determine any dose-response relationship. However, establishing such a relationship is particularly challenging due to high interindividual variability in symptom progression and neurobiological responses; there is no universal threshold or specific time point after which structural changes become detectable in all patients. The onset and extent of amygdala volume changes may differ greatly among individuals, influenced by factors such as genetic predispositions, environmental stressors, and resilience mechanisms. Therefore, larger, well-controlled longitudinal studies are needed to account for this variability and to better understand the temporal dynamics of amygdala alterations in relation to symptom duration.

Moreover, a meta-analysis examining differences in amygdala volume based on medication status revealed that the volume of the amygdala was smaller in unmedicated patients with MDD compared to HCs, whereas medicated participants exhibited increased amygdala volume compared to HCs [43]. Antidepressant treatment is believed to ameliorate brain functional abnormalities by promoting the production of neurotrophic factors and preventing glucocorticoid levels from reaching toxic levels. However, recent studies have not reported specific differences in amygdala nuclei volumes between individuals with MDD with or without antidepressant treatment [22,23]. Our analysis found no significant impact of prior medication on amygdala subfield volumes. However, variations in medication type, dosage, and duration across studies may have influenced our results. Without systematically recording the timing of medication in our study, it is possible that our patients did not receive antidepressants for a sufficient duration or dosage to affect brain structure, especially considering they underwent cMRI shortly after prescription. Further studies are needed to clarify the effects of antidepressant use on amygdala structure.

Research suggests that psychotherapeutic interventions may influence amygdala activation [39]. Manthey et al. [39], in their systematic review of 12 PTSD studies, found mixed results regarding changes in amygdala activation following psychotherapy: some studies reported decreased activation (n = 3), one showed increased activation (n = 1), and most found no significant change (n = 8). These mixed findings indicate that psychotherapy can have variable effects on amygdala function in PTSD patients. In our study, although we did not directly measure pre- and post-psychotherapy changes in amygdala activation or volume, we included prior psychotherapeutic experience as a covariate in our analyses. This decision was informed by Manthey et al.’s findings, as prior psychotherapy could potentially affect amygdala nuclei volumes and thus influence our results. By accounting for previous psychotherapeutic interventions, we aimed to control for their potential impact on amygdala structure. Our exploratory analyses did not reveal significant associations between prior psychotherapy and amygdala nuclei volumes. However, it’s important to note that we operationalized prior psychotherapy as a binary variable (yes/no), which may not capture the complexity of psychotherapeutic interventions, such as differences in therapy type, duration, or intensity. Therefore, while we attempted to model the potential influence of psychotherapy based on Manthey et al.’s review, the lack of significant findings suggests that more detailed assessments are necessary. Future studies should empirically investigate how specific aspects of psychotherapy impact amygdala nuclei volumes to better understand this relationship.

### 5.1. Limitations

Our findings should be interpreted in light of several limitations. A key limitation is the absence of a HC group. Our study represents an initial step toward precision psychiatry, aiming to determine whether different stress-related mental disorders—such as PTSD and MDD—exhibit significant differences in amygdala nuclei volumes using routine clinical cMRI data. However, without an HC group, it remains uncertain whether the observed amygdala nuclei volumes truly reflect atrophy or a lack of enlargement typically seen in HCs. Typically, multiple HC groups (including trauma-exposed and trauma-unexposed individuals) are crucial for establishing causality in cross-sectional studies [39,40]. The lack of an HC group in our study reflects common clinical practice, where such controls are not readily available. We included patients with AdjD, hypothesizing that their typically shorter duration and lower stress exposure might lead to less pronounced volume differences in the basolateral and medial nuclei compared to PTSD and MDD. However, given the absence of significant differences, it is unclear whether this indicates genuinely smaller amygdala volumes or simply a lack of atrophy. Future research should replicate our findings with multiple HC groups to clarify this issue.

Due to data privacy regulations, we did not systematically assess stress levels, trauma severity, or childhood trauma experiences—factors that could have provided valuable insights into the relationship between stress exposure and amygdala nuclei volumes [39,44]. Meta-analyses have shown differences in amygdala volumes between individuals with childhood trauma and those experiencing trauma for the first time in adulthood, suggesting that future studies should incorporate these assessments to better understand their impact on amygdala nuclei [45]. Additionally, the absence of standardized measures for self-reported symptom severity in our study limits our ability to explore its potential correlations with amygdala volumes. Although findings in the literature are mixed—with some studies indicating small effect sizes (0.02 to 0.15) and others showing no significant associations (PTSD: [15]; MDD: [20,21])—the lack of such data in our sample restricts a more nuanced interpretation of our results, warranting further investigation in future research.

An important aspect of our study was the inclusion of age and gender as covariates, given their known influence on amygdala volumes. By controlling for these factors in our analyses, we aimed to isolate the effects of the psychiatric conditions. Our adjusted results continued to show no significant differences in amygdala nuclei volumes among the diagnostic groups when accounting for age and gender. This suggests that demographic variations did not confound our findings. Future research with larger samples may further investigate subtle effects of age and gender on amygdala structure.

Another limitation is that comorbid personality disorders were not excluded from our study. Personality disorders, such as Borderline Personality Disorder (BPD), have been associated with structural brain volume differences in the amygdala. In our sample, two participants had a secondary diagnosis of BPD—one with PTSD without MDD and one with MDD. To assess the potential impact, we repeated our analyses excluding these participants and found no significant differences in the results. This suggests that their inclusion did not substantially affect our findings. However, future studies should consider excluding or separately analyzing participants with personality disorders to better understand their specific impact on amygdala nuclei volumes.

Moreover, we did not systematically document the specific types of trauma experienced by our participants. Although it is likely that most PTSD patients in our sample experienced military-related trauma, as our sample consisted solely of soldiers, this remains speculative without empirical verification. Given that different types of trauma may differentially affect amygdala nuclei, future studies should incorporate detailed trauma history assessments.

Moreover, our study focused solely on the amygdala and did not examine other brain regions implicated in PTSD, MDD, and AdjD, such as the insula, anterior cingulate cortex (ACC), and hippocampus. Additionally, we did not assess the connectivity between these regions, which is crucial for understanding the neural networks underlying these disorders. Functional connectivity analyses, particularly using functional MRI (fMRI), can provide valuable insights into how these regions interact and contribute to symptomatology [12]. Future research should adopt a more comprehensive approach by including these regions and examining their connectivity, as fMRI studies may offer more detailed information than structural cMRI scans in elucidating the complex neural mechanisms underlying these disorders.

Furthermore, certain confounding factors were not fully controlled for in our study. Specifically, some participants had comorbid personality disorders, and although excluding them did not alter our results (see S5 Table), their presence may still influence brain structure. Additionally, we did not systematically differentiate between Type 1 and Type 2 trauma or assess whiplash injuries, both of which are common in military populations and may affect brain structure. These limitations may have introduced bias into our results, potentially affecting their validity.

The interpretation of symptom duration in this study may be limited due to the absence of a more detailed investigation into symptom onset. Specifically, standardized assessment tools such as the Clinician-Administered PTSD Scale (CAPS) were not utilized, which could have facilitated a more comprehensive evaluation and analysis of symptomatology. Without these tools, the ability to precisely determine the onset and progression of symptoms is constrained. Consequently, the findings regarding symptom duration should be interpreted with caution, and future research should consider incorporating standardized assessments to enhance the accuracy and depth of symptom analysis.

Lastly, our sample had significantly fewer women than men, which is typical in military samples but may have influenced our results. Future research should consider more balanced gender representation to enhance generalizability.

## 6. Conclusion

In summary, our study examined amygdala nuclei volumes among patients with various mental disorders, including MDD, PTSD without MDD (PTSD-MDD), comorbid PTSD with MDD (PTSD+MDD), and AdjD. While we did not find significant differences in amygdala nuclei volumes among these groups, the study addresses an important issue by focusing on a rarely studied subset of patient groups. This research contributes to the literature by highlighting the complexities and nuances involved in understanding structural brain differences in these mental health conditions.

Although models like that of Shalev et al. [6] suggest that the basolateral complex of the amygdala may be associated with specific PTSD symptoms, such as fear conditioning and extinction reconsolidation, our study did not find any significant volumetric differences in the basolateral complex between PTSD, MDD, and AdjD. This lack of differentiation in amygdala subfield volumes raises questions about the applicability of structural neuroimaging as a standalone tool for distinguishing between these disorders in clinical settings. It may indicate that while the basolateral amygdala is functionally involved in PTSD symptomatology, structural differences alone may not sufficiently capture the nuanced neurobiological distinctions between PTSD, MDD, and AdjD. This underscores the importance of including multiple control groups, such as trauma-exposed and trauma-unexposed healthy controls, in future research to better determine whether volume differences in amygdala nuclei are disorder-specific or reflect broader stress-related mechanisms.

## Supporting information

S1 Table Results of ANCOVA parameters with and without bootstrapping. (DOCX)

S2 Table Results of ANCOVA with and without outliers. (DOCX)

S3 Table Overview of ANCOVA results comparing recurrent vs. first-onset depressive episodes and adjustment disorder duration less than 6 months vs. greater than 6 months. (DOCX)

S4 Table Overview of ANCOVA results comparing mild, moderate and serve depressive episodes. (DOCX)

S5 Table Overview of ANCOVA results excluding participants with comorbid personality disorders. (DOCX)
