# Supplementary material for: Investigating amygdala nuclei volumes in military personnel with post-traumatic stress disorder, major depressive disorder, and adjustment disorder: A retrospective cross-sectional study using clinical routine data
Source: PLoS One. 2025 Jan 16;20(1):e0317573. doi: 10.1371/journal.pone.0317573 (PMC11737849; doi:10.1371/journal.pone.0317573)
Supplement: S1 Table — (DOCX) [file pone.0317573.s001.docx]

Supplementary Table 1**.** *Results of ANCOVA parameters with and without bootstrapping*

|  |  | Basal nucleus | | | | |  | Lateral nucleus | | | | |  | Accessory basal nucleus | | | | |  | Medial nucleus | | | | |
| --- | --- | --- | --- | --- | --- | --- | --- | --- | --- | --- | --- | --- | --- | --- | --- | --- | --- | --- | --- | --- | --- | --- | --- | --- |
|  |  | *b* |  | 95% CI | | |  | *b* |  | 95% CI | | |  | *b* |  | 95% CI | | |  | *b* |  | 95% CI | | |
|  |  | ANCOVA parameters without bootstrapping | | | | | | | | | | | | | | | | | | | | | | |
| Intercept |  | 284.0 |  | [143.7 | ; | 424.3] |  | 327.9 |  | [131.7 | ; | 524.1] |  | 169.9 |  | [81.5 | ; | 258.3] | - | 2.0 |  | [-18.7 | ; | 14.7] |
| eTIV |  | 0.0004 |  | [0.0003 | ; | 0.001] |  | 0.001 |  | [0.001 | ; | 0.001] |  | 0.0002 |  | [0.0002 | ; | 0.0003] |  | 0.00003 |  | [0,00002 | ; | 0,00004] |
| Age | - | 1.17 |  | [-2.5 | ; | 0.1] | - | 0.31 |  | [-2.2 | ; | 1.5] | - | 0.96 |  | [-1.8 | ; | -0.1] | - | 0.1 |  | [-0.2 | ; | 0.1] |
| Gender |  | 71.6 |  | [33.3 | ; | 109.9] |  | 119.7 |  | [66.1 | ; | 173.2] |  | 35.5 |  | [11.3 | ; | 59.6] |  | 0.6 |  | [-3.9 | ; | 5.1] |
| Patient group  (references group: AD) |  |  |  |  |  |  |  |  |  |  |  |  |  |  |  |  |  |  |  |  |  |  |  |  |
| MDD |  | 5.06 |  | [-29.0 | ; | 39.1] |  | 18.6 |  | [-29.0 | ; | 66.3] | - | 6.8 |  | [-28.3 | ; | 14.6] | - | 2.1 |  | [-6.2 | ; | 1.8] |
| PTSD |  | 3.31 |  | [-33.0 | ; | 39.6] |  | 21.7 |  | [-29.1 | ; | 72.5] | - | 7.9 |  | [-30.9 | ; | 14.9] | - | 3.2 |  | [-7.5 | ; | 1.1] |
| PTSD+MDD | - | 7.87 |  | [-46.7 | ; | 30.9] |  | 11.6 |  | [-42.7 | ; | 65.9] | - | 17.6 |  | [-42.1 | ; | 6.8] | - | 2.8 |  | [-7.4 | ; | 1.8] |
|  |  | ANCOVA parameters with bootstrapping | | | | | | | | | | | | | | | | | | | | | | |
| Intercept |  | 284.0 |  | [136.6 | ; | 441.6] |  | 327.9 |  | [117.2 | ; | 578.9] |  | 169.9 |  | [84.2 | ; | 262.0] | - | 2.0 |  | [-18.2 | ; | 12.9] |
| eTIV |  | 0.0004 |  | [0.0003 | ; | 0.001] |  | 0.001 |  | [0.001 | ; | 0.001] |  | 0.0002 |  | [0.0002 | ; | 0.0003] |  | 0.00003 |  | [0,00002 | ; | 0,00004] |
| Age | - | 1.17 |  | [-2.4 | ; | 0.3] | - | 0.31 |  | [-2.0 | ; | 1.7] | - | 0.96 |  | [-1.8 | ; | 0.1] | - | 0.1 |  | [-0.2 | ; | 0.1] |
| Gender |  | 71.6 |  | [34.2 | ; | 107.6] |  | 119.7 |  | [80.9 | ; | 157.3] |  | 35.5 |  | [12.2 | ; | 59.3] |  | 0.6 |  | [-2.8 | ; | 4.0] |
| Patient group  (references group: AD) |  |  |  |  |  |  |  |  |  |  |  |  |  |  |  |  |  |  |  |  |  |  |  |  |
| MDD |  | 5.06 |  | [-26.7 | ; | 35.9] |  | 18.6 |  | [-30.8 | ; | 67.8] | - | 6.8 |  | [-25.1 | ; | 10.4] | - | 2.1 |  | [-6.0 | ; | 1.7] |
| PTSD |  | 3.31 |  | [-26.4 | ; | 35.0] |  | 21.7 |  | [-22.3 | ; | 68.5] | - | 7.9 |  | [-30.1 | ; | 12.8] | - | 3.2 |  | [-7.1 | ; | 0.3] |
| PTSD+MDD | - | 7.87 |  | [-35.1 | ; | 18.4] |  | 11.6 |  | [-29.4 | ; | 49.6] | - | 17.6 |  | [-36.7 | ; | 1.3] | - | 2.8 |  | [-7.0 | ; | 1.2] |

*Note.* Model parameters are based on 1000 bootstrap samples.
Bias-corrected confidence intervals have been applied.
