# Supplementary material for: Investigating amygdala nuclei volumes in military personnel with post-traumatic stress disorder, major depressive disorder, and adjustment disorder: A retrospective cross-sectional study using clinical routine data
Source: PLoS One. 2025 Jan 16;20(1):e0317573. doi: 10.1371/journal.pone.0317573 (PMC11737849; doi:10.1371/journal.pone.0317573)
Supplement: S2 Table — (DOCX) [file pone.0317573.s002.docx]

Supplementary Table 2. *Results of ANCOVA with and without outliers*

|  |  | Basal nucleus | | | | | | |  | Lateral nucleus | | | | | | |  | Accessory basal nucleus | | | | | | |  | Medial nucleus | | | | | | |
| --- | --- | --- | --- | --- | --- | --- | --- | --- | --- | --- | --- | --- | --- | --- | --- | --- | --- | --- | --- | --- | --- | --- | --- | --- | --- | --- | --- | --- | --- | --- | --- | --- |
| variables |  | F | (df) |  | *p* | |  | η_p_² |  | F | (df) |  | *p* | |  | η_p_² |  | F | (df) |  | *p* | |  | η_p_² |  | F | (df) |  | *p* | |  | η_p_² |
| *Main analyses* |  |  |  |  |  | |  |  |  |  |  |  |  | |  |  |  |  |  |  |  | |  |  |  |  |  |  |  | |  |  |
| patient group |  | 0.2 | (3, 178) |  |  | .872 |  | .004 |  | 0.2 | (2, 178) |  |  | .837 |  | .005 |  | 0.7 | (3, 178) |  |  | .544 |  | 0.12 |  | 0.7 | (3, 178) |  |  | .518 |  | .013 |
| *Explorative analyses* |  |  |  |  |  |  |  |  |  |  |  |  |  |  |  |  |  |  |  |  |  |  |  |  |  |  |  |  |  |  |  |  |
| symptom duration |  | 1.0 | (1, 141) |  |  | .310 |  | .007 |  | 0.6 | (1, 141) |  |  | .414 |  | .005 |  | 1.7 | (1, 141) |  |  | .183 |  | .013 |  | 0.3 | (1, 141) |  |  | .556 |  | .002 |
| symptom duration*^*^*patient group |  | 0.9 | (3, 141) |  |  | .403 |  | .020 |  | 1.9 | (3, 141) |  |  | .132 |  | .039 |  | 0.6 | (3, 141) |  |  | .607 |  | .013 |  | 0.4 | (3, 141) |  |  | .706 |  | .010 |
| medication |  | 0.3 | (1, 141) |  |  | .543 |  | .003 |  | 0.1 | (1, 141) |  |  | .788 |  | .001 |  | 0.2 | (1, 141) |  |  | .596 |  | .002 |  | 0.1 | (1, 141) |  |  | .772 |  | .001 |
| medication*^*^*patient group |  | 0.9 | (3, 141) |  |  | .426 |  | .020 |  | 0.1 | (3, 141) |  |  | .930 |  | .003 |  | 1.0 | (3, 141) |  |  | .352 |  | .023 |  | 0.9 | (3, 141) |  |  | .416 |  | .020 |
| pre psychotherapy |  | 0.6 | (1, 141) |  |  | .420 |  | .005 |  | 0.1 | (1, 141) |  |  | .671 |  | .001 |  | 1.2 | (1, 141) |  |  | .257 |  | .009 |  | 3.7 | (1, 141) |  |  | .055 |  | .026 |
| pre psychotherapy*^*^*patient group |  | 0.1 | (3, 141) |  |  | .924 |  | .003 |  | 0.4 | (3, 141) |  |  | .726 |  | .009 |  | 0.6 | (3, 141) |  |  | .615 |  | .013 |  | 0.5 | (3, 141) |  |  | .665 |  | .011 |
| *Controlling for* |  |  |  |  |  |  |  |  |  |  |  |  |  |  |  |  |  |  |  |  |  |  |  |  |  |  |  |  |  |  |  |  |
| eTIV |  | 69.0 | (1, 178) |  | < | .001 |  | .280 |  | 75.0 | (1, 178) |  | < | .001 |  | .297 |  | 76.0 | (1, 178) |  | < | .001 |  | .299 |  | 41.4 | (1, 178) |  | < | .001 |  | .189 |
| Age |  | 2.9 | (1, 178) |  |  | .090 |  | .016 |  | 0.1 | (1, 178) |  |  | .745 |  | .001 |  | 4.9 | (1, 178) |  |  | .027 |  | .027 |  | 1.7 | (1, 178) |  |  | .185 |  | .010 |
| Gender |  | 13.6 | (1, 178) |  | < | .001 |  | .071 |  | 19.4 | (1, 178) |  | < | .001 |  | .099 |  | 8.4 | (1, 178) |  |  | .004 |  | .045 |  | 0.1 | (1, 178) |  |  | .795 |  | .001 |
|  |  | *without outliers* | | | | | | | | | | | | | | | | | | | | | | | | | | | | | | |
| *Main analyses* |  |  |  |  |  | |  |  |  |  |  |  |  | |  |  |  |  |  |  |  | |  |  |  |  |  |  |  | |  |  |
| patient group |  | 0.2 | (3, 174) |  |  | .852 |  | .005 |  | 0.2 | (2, 175) |  |  | .636 |  | .010 |  | 0.7 | (3, 175) |  |  | .500 |  | 0.13 |  | 1.2 | (3, 175) |  |  | .277 |  | .022 |
| *Explorative analyses* |  |  |  |  |  |  |  |  |  |  |  |  |  |  |  |  |  |  |  |  |  |  |  |  |  |  |  |  |  |  |  |  |
| symptom duration |  | 1.1 | (1, 139) |  |  | .295 |  | .008 |  | 0.7 | (1, 139) |  |  | .389 |  | .005 |  | 1.8 | (1, 139) |  |  | .178 |  | .013 |  | 0.2 | (1, 139) |  |  | .654 |  | .001 |
| symptom duration*^*^*patient group |  | 0.8 | (3, 139) |  |  | .485 |  | .017 |  | 1.7 | (3, 139) |  |  | .156 |  | .037 |  | 0.5 | (3, 139) |  |  | .671 |  | .011 |  | 0.5 | (3, 139) |  |  | .674 |  | .011 |
| medication |  | 0.2 | (1, 139) |  |  | .644 |  | .002 |  | 0.1 | (1, 139) |  |  | .991 |  | .001 |  | 0.2 | (1, 139) |  |  | .589 |  | .002 |  | 0.1 | (1, 139) |  |  | .865 |  | .001 |
| medication*^*^*patient group |  | 0.9 | (3, 139) |  |  | .426 |  | .020 |  | 0.1 | (3, 139) |  |  | .996 |  | .001 |  | 1.0 | (3, 139) |  |  | .382 |  | .022 |  | 0.7 | (3, 139) |  |  | .536 |  | .016 |
| pre psychotherapy |  | 0.2 | (1, 139) |  |  | .624 |  | .002 |  | 0.1 | (1, 139) |  |  | .962 |  | .001 |  | 1.1 | (1, 139) |  |  | .285 |  | .008 |  | 3.4 | (1, 139) |  |  | .066 |  | .024 |
| pre psychotherapy*^*^*patient group |  | 0.1 | (3, 139) |  |  | .922 |  | .003 |  | 0.1 | (3, 139) |  |  | .898 |  | .004 |  | 0.6 | (3, 139) |  |  | .602 |  | .013 |  | 0.4 | (3, 139) |  |  | .697 |  | .010 |
| *Controlling for* |  |  |  |  |  |  |  |  |  |  |  |  |  |  |  |  |  |  |  |  |  |  |  |  |  |  |  |  |  |  |  |  |
| eTIV |  | 53.9 | (1, 174) |  | < | .001 |  | .237 |  | 61.9 | (1, 175) |  | < | .001 |  | .262 |  | 68.4 | (1, 175) |  | < | .001 |  | .281 |  | 41.4 | (1, 175) |  | < | .001 |  | .184 |
| Age |  | 3.3 | (1, 174) |  |  | .071 |  | .019 |  | 0.1 | (1, 175) |  |  | .745 |  | .001 |  | 5.8 | (1, 175) |  |  | .017 |  | .032 |  | 1.7 | (1, 175) |  |  | .099 |  | .015 |
| Gender |  | 11.0 | (1, 174) |  |  | .001 |  | .060 |  | 16.5 | (1, 175) |  | < | .001 |  | .087 |  | 8.6 | (1, 175) |  |  | .004 |  | .047 |  | 0.1 | (1, 175) |  |  | .838 |  | .001 |

*Note.* eTIV = estimated intracranial volume, pre psychotherapy = pretreatment psychotherapeutic.
