# Supplementary material for: Investigating amygdala nuclei volumes in military personnel with post-traumatic stress disorder, major depressive disorder, and adjustment disorder: A retrospective cross-sectional study using clinical routine data
Source: PLoS One. 2025 Jan 16;20(1):e0317573. doi: 10.1371/journal.pone.0317573 (PMC11737849; doi:10.1371/journal.pone.0317573)
Supplement: S3 Table — (DOCX) [file pone.0317573.s003.docx]

Supplementary Table 3: *Overview of ANCOVA results comparing recurrent vs. first-onset depressive episodes and adjustment disorder duration less than 6 months vs. greater than 6 months.*

|  |  | Basal nucleus | | | | | | |  | Lateral nucleus | | | | | | |  | Accessory basal nucleus | | | | | | |  | Medial nucleus | | | | | | |
| --- | --- | --- | --- | --- | --- | --- | --- | --- | --- | --- | --- | --- | --- | --- | --- | --- | --- | --- | --- | --- | --- | --- | --- | --- | --- | --- | --- | --- | --- | --- | --- | --- |
| variables |  | F | (df) |  | *p* | |  | η_p_² |  | F | (df) |  | *p* | |  | η_p_² |  | F | (df) |  | *p* | |  | η_p_² |  | F | (df) |  | *p* | |  | η_p_² |
| *Main analyses* |  |  |  |  |  | |  |  |  |  |  |  |  | |  |  |  |  |  |  |  | |  |  |  |  |  |  |  | |  |  |
| patient group |  | 0.8 | (5, 176) |  |  | .550 |  | .022 |  | 0.9 | (5, 176) |  |  | .479 |  | .025 |  | 1.0 | (5, 176) |  |  | .367 |  | 0.03 |  | 0.5 | (5, 176) |  |  | .728 |  | .016 |
| *Explorative analyses* |  |  |  |  |  |  |  |  |  |  |  |  |  |  |  |  |  |  |  |  |  |  |  |  |  |  |  |  |  |  |  |  |
| symptom duration |  | 1.3 | (1, 134) |  |  | .246 |  | .010 |  | 5.0 | (1, 134) |  |  | .026 |  | .037 |  | 0.2 | (1, 134) |  |  | .591 |  | .002 |  | 2.7 | (1, 134) |  |  | .097 |  | .020 |
| symptom duration*^*^*patient group |  | 1.3 | (4, 134) |  |  | .231 |  | .049 |  | 2.8 | (4, 134) |  |  | .018 |  | .096 |  | 0.5 | (4, 134) |  |  | .582 |  | .027 |  | 1.2 | (4, 134) |  |  | .309 |  | .043 |
| medication |  | 0.1 | (1, 134) |  |  | .827 |  | .001 |  | 0.1 | (1, 134) |  |  | .892 |  | .001 |  | 0.7 | (1, 134) |  |  | .740 |  | .001 |  | 0.4 | (1, 134) |  |  | .827 |  | .001 |
| medication*^*^*patient group |  | 1.3 | (4, 134) |  |  | .269 |  | .038 |  | 1.5 | (4, 134) |  |  | .187 |  | .045 |  | 0.2 | (4, 134) |  |  | .235 |  | .040 |  | 1.7 | (4, 134) |  |  | .139 |  | .050 |
| pre psychotherapy |  | 0.8 | (1, 134) |  |  | .360 |  | .006 |  | 0.1 | (1, 134) |  |  | .670 |  | .001 |  | 0.3 | (1, 134) |  |  | .372 |  | .006 |  | 3.9 | (1, 134) |  |  | .050 |  | .028 |
| pre psychotherapy*^*^*patient group |  | 0.1 | (4, 134) |  |  | .969 |  | .004 |  | 0.3 | (4, 134) |  |  | .871 |  | .009 |  | 0.9 | (4, 134) |  |  | .901 |  | .008 |  | 0.7 | (4, 134) |  |  | .565 |  | .022 |
| *Controlling for* |  |  |  |  |  |  |  |  |  |  |  |  |  |  |  |  |  |  |  |  |  |  |  |  |  |  |  |  |  |  |  |  |
| eTIV |  | 69.9 | (1, 176) |  | < | .001 |  | .285 |  | 75.0 | (1, 176) |  | < | .001 |  | .305 |  | 77.1 | (1, 176) |  | < | .001 |  | .305 |  | 41.5 | (1, 176) |  | < | .001 |  | .191 |
| Age |  | 3.1 | (1, 176) |  |  | .090 |  | .018 |  | 0.1 | (1, 176) |  |  | .671 |  | .001 |  | 5.2 | (1, 176) |  |  | .023 |  | .029 |  | 1.8 | (1, 176) |  |  | .176 |  | .010 |
| Gender |  | 12.8 | (1, 176) |  | < | .001 |  | .068 |  | 19.4 | (1, 176) |  | < | .001 |  | .101 |  | 7.8 | (1, 176) |  |  | .006 |  | .043 |  | 0.1 | (1, 176) |  |  | .760 |  | .001 |

*Note.* eTIV = estimated intracranial volume, pre psychotherapy = pretreatment psychotherapeutic. The patient group factor consists of: First-onset depressive episode: N = 41; Recurrent depressive episode: N = 29; PTSD without MDD: N = 42; PTSD with MDD: N = 31; Adjustment Disorder with symptom duration < 6 months: N = 13 and Adjustment Disorder with symptom duration > 6 months: N = 14.
