# Supplementary material for: Investigating amygdala nuclei volumes in military personnel with post-traumatic stress disorder, major depressive disorder, and adjustment disorder: A retrospective cross-sectional study using clinical routine data
Source: PLoS One. 2025 Jan 16;20(1):e0317573. doi: 10.1371/journal.pone.0317573 (PMC11737849; doi:10.1371/journal.pone.0317573)
Supplement: S4 Table — (DOCX) [file pone.0317573.s004.docx]

Supplementary Table 4: *Overview of ANCOVA results comparing mild, moderate and serve depressive episodes.*

|  |  | Basal nucleus | | | | | | |  | Lateral nucleus | | | | | | |  | Accessory basal nucleus | | | | | | |  | Medial nucleus | | | | | | |
| --- | --- | --- | --- | --- | --- | --- | --- | --- | --- | --- | --- | --- | --- | --- | --- | --- | --- | --- | --- | --- | --- | --- | --- | --- | --- | --- | --- | --- | --- | --- | --- | --- |
| variables |  | F | (df) |  | *p* | |  | η_p_² |  | F | (df) |  | *p* | |  | η_p_² |  | F | (df) |  | *p* | |  | η_p_² |  | F | (df) |  | *p* | |  | η_p_² |
| *Main analyses* |  |  |  |  |  | |  |  |  |  |  |  |  | |  |  |  |  |  |  |  | |  |  |  |  |  |  |  | |  |  |
| patient group |  | 0.2 | (5, 176) |  |  | .941 |  | .007 |  | 0.5 | (5, 176) |  |  | .744 |  | .015 |  | 0.5 | (5, 176) |  |  | .750 |  | 0.01 |  | 0.8 | (5, 176) |  |  | .524 |  | .023 |
| *Explorative analyses* |  |  |  |  |  |  |  |  |  |  |  |  |  |  |  |  |  |  |  |  |  |  |  |  |  |  |  |  |  |  |  |  |
| symptom duration |  | 1.2 | (1, 133) |  |  | .260 |  | .010 |  | 4.9 | (1, 133) |  |  | .027 |  | .036 |  | 0.1 | (1, 133) |  |  | .693 |  | .001 |  | 0.1 | (1, 133) |  |  | .687 |  | .001 |
| symptom duration*^*^*patient group |  | 0.9 | (5, 133) |  |  | .458 |  | .034 |  | 1.6 | (5, 133) |  |  | .154 |  | .058 |  | 0.5 | (5, 133) |  |  | .718 |  | .021 |  | 0.3 | (5, 133) |  |  | .875 |  | .013 |
| medication |  | 1.0 | (1, 133) |  |  | .316 |  | .008 |  | 1.5 | (1, 133) |  |  | .222 |  | .011 |  | 0.2 | (1, 133) |  |  | .653 |  | .002 |  | 0.2 | (1, 133) |  |  | .631 |  | .002 |
| medication*^*^*patient group |  | 1.7 | (5, 133) |  |  | .130 |  | .061 |  | 1.1 | (5, 133) |  |  | .355 |  | .040 |  | 1.1 | (5, 133) |  |  | .350 |  | .041 |  | 0.5 | (5, 133) |  |  | .730 |  | .021 |
| pre psychotherapy |  | 0.2 | (1, 133) |  |  | .865 |  | .001 |  | 0.2 | (1, 133) |  |  | .611 |  | .002 |  | 0.5 | (1, 133) |  |  | .469 |  | .004 |  | 1.2 | (1, 133) |  |  | .272 |  | .009 |
| pre psychotherapy*^*^*patient group |  | 0.5 | (5, 133) |  |  | .743 |  | .020 |  | 0.5 | (5, 133) |  |  | .749 |  | .020 |  | 0.8 | (5, 133) |  |  | .520 |  | .031 |  | 0.2 | (5, 133) |  |  | .919 |  | .011 |
| *Controlling for* |  |  |  |  |  |  |  |  |  |  |  |  |  |  |  |  |  |  |  |  |  |  |  |  |  |  |  |  |  |  |  |  |
| eTIV |  | 69.0 | (1, 176) |  | < | .001 |  | .280 |  | 75.3 | (1, 176) |  | < | .001 |  | .300 |  | 75.3 | (1, 176) |  | < | .001 |  | .300 |  | 41.6 | (1, 176) |  | < | .001 |  | .191 |
| Age |  | 2.9 | (1, 176) |  |  | .081 |  | .017 |  | 0.2 | (1, 176) |  |  | .603 |  | .002 |  | 5.1 | (1, 176) |  |  | .024 |  | .028 |  | 2.2 | (1, 176) |  |  | .136 |  | .013 |
| Gender |  | 13.6 | (1, 176) |  | < | .001 |  | .070 |  | 18.5 | (1, 176) |  | < | .001 |  | .095 |  | 8.0 | (1, 176) |  |  | .005 |  | .044 |  | 0.2 | (1, 176) |  |  | .864 |  | .001 |

*Note.* eTIV = estimated intracranial volume, pre psychotherapy = pretreatment psychotherapeutic. The patient group factor consists of: Mild depressive episode: N = 5; Moderate depressive episode: N = 37; Severe depressive episode: N = 21; PTSD without MDD: N = 42; PTSD with MDD: N = 31; Adjustment Disorder (AdjD): N = 24.
