# Supplementary material for: Investigating amygdala nuclei volumes in military personnel with post-traumatic stress disorder, major depressive disorder, and adjustment disorder: A retrospective cross-sectional study using clinical routine data
Source: PLoS One. 2025 Jan 16;20(1):e0317573. doi: 10.1371/journal.pone.0317573 (PMC11737849; doi:10.1371/journal.pone.0317573)
Supplement: S5 Table — (DOCX) [file pone.0317573.s005.docx]

Supplementary Table 5: *Overview of ANCOVA Results Excluding Participants with Comorbid Personality Disorders*.

|  |  | Basal nucleus | | | | | | |  | Lateral nucleus | | | | | | |  | Accessory basal nucleus | | | | | | |  | Medial nucleus | | | | | | |
| --- | --- | --- | --- | --- | --- | --- | --- | --- | --- | --- | --- | --- | --- | --- | --- | --- | --- | --- | --- | --- | --- | --- | --- | --- | --- | --- | --- | --- | --- | --- | --- | --- |
| variables |  | F | (df) |  | *p* | |  | η_p_² |  | F | (df) |  | *p* | |  | η_p_² |  | F | (df) |  | *p* | |  | η_p_² |  | F | (df) |  | *p* | |  | η_p_² |
| *Main analyses* |  |  |  |  |  | |  |  |  |  |  |  |  | |  |  |  |  |  |  |  | |  |  |  |  |  |  |  | |  |  |
| patient group |  | 0.4 | (3, 166) |  |  | .708 |  | .008 |  | 0.4 | (3, 166) |  |  | .712 |  | .008 |  | 0.9 | (3, 166) |  |  | .438 |  | 0.16 |  | 0.5 | (5, 176) |  |  | .728 |  | .016 |
| *Explorative analyses* |  |  |  |  |  |  |  |  |  |  |  |  |  |  |  |  |  |  |  |  |  |  |  |  |  |  |  |  |  |  |  |  |
| symptom duration |  | 0.5 | (1, 130) |  |  | .455 |  | .004 |  | 0.9 | (1, 130) |  |  | .326 |  | .007 |  | 1.4 | (1, 130) |  |  | .226 |  | .011 |  | 0.1 | (1, 130) |  |  | .714 |  | .001 |
| symptom duration*^*^*patient group |  | 1.2 | (3, 130) |  |  | .299 |  | .028 |  | 1.9 | (3, 130) |  |  | .120 |  | .044 |  | 0.6 | (3, 130) |  |  | .570 |  | .015 |  | 0.6 | (3, 130) |  |  | .608 |  | .014 |
| medication |  | 0.3 | (1, 130) |  |  | .546 |  | .003 |  | 0.1 | (1, 130) |  |  | .877 |  | .001 |  | 0.2 | (1, 130) |  |  | .637 |  | .002 |  | 0.1 | (1, 130) |  |  | .802 |  | .001 |
| medication*^*^*patient group |  | 1.0 | (3, 130) |  |  | .395 |  | .023 |  | 0.2 | (3, 130) |  |  | .829 |  | .007 |  | 1.0 | (3, 130) |  |  | .381 |  | .023 |  | 1.1 | (3, 130) |  |  | .340 |  | .025 |
| pre psychotherapy |  | 0.2 | (1, 130) |  |  | .637 |  | .002 |  | 0.1 | (1, 130) |  |  | .838 |  | .001 |  | 0.6 | (1, 130) |  |  | .410 |  | .005 |  | 2.9 | (1, 130) |  |  | .090 |  | .022 |
| pre psychotherapy*^*^*patient group |  | 0.4 | (3, 130) |  |  | .706 |  | .011 |  | 0.6 | (3, 130) |  |  | .587 |  | .015 |  | 1.1 | (3, 130) |  |  | .344 |  | .025 |  | 0.4 | (3, 130) |  |  | .688 |  | .011 |
| *Controlling for* |  |  |  |  |  |  |  |  |  |  |  |  |  |  |  |  |  |  |  |  |  |  |  |  |  |  |  |  |  |  |  |  |
| eTIV |  | 68.4 | (1, 166) |  | < | .001 |  | .292 |  | 72.4 | (1, 176) |  | < | .001 |  | .304 |  | 76.4 | (1, 166) |  | < | .001 |  | .315 |  | 41.5 | (1, 176) |  | < | .001 |  | .191 |
| Age |  | 4.0 | (1, 166) |  |  | .046 |  | .024 |  | 0.2 | (1, 176) |  |  | .587 |  | .002 |  | 6.6 | (1, 166) |  |  | .011 |  | .038 |  | 1.8 | (1, 176) |  |  | .176 |  | .010 |
| Gender |  | 18.2 | (1, 166) |  |  | .001 |  | .099 |  | 20.8 | (1, 176) |  | < | .001 |  | .112 |  | 10.9 | (1, 166) |  |  | .001 |  | .062 |  | 0.1 | (1, 176) |  |  | .760 |  | .001 |

*Note.* eTIV = estimated intracranial volume, pre psychotherapy = pretreatment psychotherapeutic.
